# Tailor-Made Surgical Strategy for Coexistent Cervical Myelopathy and Bilateral Radiculopathy. Combined Laminoplasty, Laminectomy, and Foraminotomy: Report of Two Cases

**DOI:** 10.7759/cureus.50195

**Published:** 2023-12-08

**Authors:** Yoshinori Maki, Toshinari Kawasaki, Motohiro Takayama

**Affiliations:** 1 Neurosurgery, Hikone Chuo Hospital, Hikone, JPN

**Keywords:** foraminotomy, laminectomy, laminoplasty, radiculopathy, myelopathy, cervical spondylosis

## Abstract

*Cervical spondylosis* is a common and degenerative disease consisting of myelopathy and radiculopathy. Surgical treatment can be considered for patients with cervical spondylosis resulting in severe pain, motor weakness, ambulation difficulty, and urination disorder. As myelopathy and radiculopathy often coexist, two-staged anterior and posterior fixation/decompression surgery can be selected to resolve those two pathologies. However, due to the invasiveness of that management, posterior surgery in a single session seems favorable. In this study, we present two cases of cervical spondylosis. A 67-year-old man was complaining of pain in the neck and right upper extremity. Radiographically, cervical canal stenosis was concurrently diagnosed with the foraminal stenosis of the left C3/4 and right C6/7. Laminoplasty from C3 to C6 (left open; C3 to C5, right open; C6) and foraminotomy of the left C3/4 and right C6/7 were performed in a single session. Another 43-year-old man was bothered by pain in the neck and bilateral upper extremities resulting from cervical canal stenosis and bilateral foraminal stenosis of C6/7. Laminoplasty from C3 to C5, laminectomy of C6, and foraminotomy of bilateral C6/7 were performed in a single session. Preoperative symptoms were remitted in both cases. As described in our cases, a tailor-made combination of laminoplasty, laminectomy, and foraminotomy can effectively resolve cervical spondylosis in a single session.

## Introduction

*Cervical spondylosis* is a common disease caused by degenerative intervertebral discs and ligaments and osteophyte formation. This disease can result in myelopathy and radiculopathy manifesting symptoms such as sensory disturbance and motor weakness in the neck, shoulder, and upper/lower extremities, impaired ambulation, and dysuria [1,2]. Only medication or rehabilitation therapy can be considered for patients with non-severe symptoms related to cervical spondylosis [2]. Meanwhile, surgical management can be considered for patients with cervical spondylosis resistant to non-invasive management [3,4].

The diagnosis of myelopathy and radiculopathy based on neurological examination is essential to resolve cervical spondylosis symptoms surgically. However, distinguishing myelopathy and radiculopathy is complicated, as these often coexist [5]. Combined anterior and posterior approaches in two sessions can typically performed for cervical kyphosis, but this surgical strategy can also be considered for coexitent myelopathy and radiculopathy [5,6]. Due to the invasiveness of this strategy, we proposed combining laminoplasty and foraminotomy in a single session [5]. This method, where myelopathy and radiculopathy can be simultaneously resolved, is advantageous if the radiculopathy exists ipsilaterally to the side where spacers for laminoplasty are inserted. However, in patients with myelopathy coexistent with bilateral and/or multi-level radiculopathy, this approach has its limitations.

Herein, we describe two cases of cervical spondylosis resulting in coexistent myelopathy and bilateral or multi-level radiculopathy treated by posterior approach in a single session. In this report, we highlight the importance of a tailor-made surgical strategy of combined laminoplasty, laminectomy, and foraminotomy for coexistent cervical myelopathy and radiculopathy.

## Case presentation

Case 1

**Figure 1:**
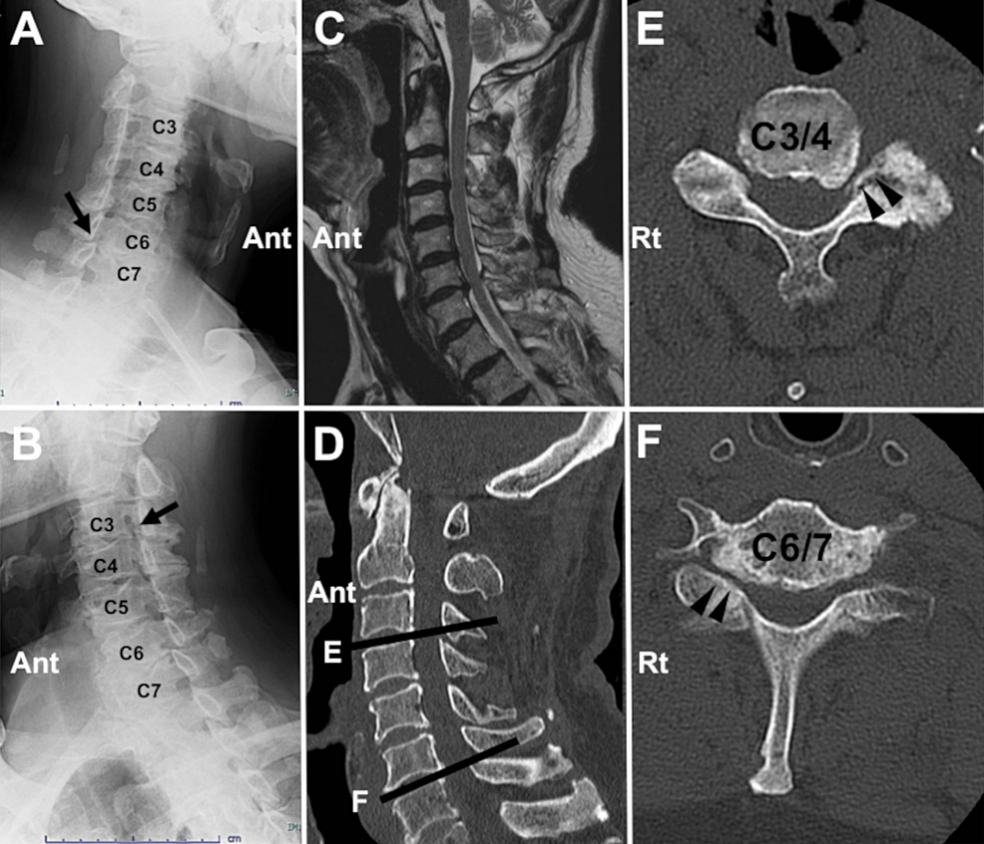
Preoperative radiographic images (Case 1). Oblique X-ray images reveal the stenosis in the right C6/7 foramen (A, black arrow) and in the left C3/4 foramen (B, black arrow). A sagittal magnetic resonance image shows cervical spinal canal stenosis (C). (D-E) Computed tomography images disclose osteophytes forming the foraminal stenosis in the left C3/4 (E) and right C6/7 (F) levels (Ant: anterior, Rt: right).

A 67-year-old man presented to our neurosurgery department complaining of neck stiffness on the left side and pain radiating to the right upper extremity. The region of the sensory disturbance approximately corresponded to the dermatome of C7. He was right-handed; however, the grip strength of the right hand was lower (31.3kg) than that of the left hand (34.4kg). He also mentioned that he suffered from neck pain when he kept an extended position of the neck. The neck pain increased gradually, even with the normal neck position. The visual analogue scale (VAS) scores of the pain in the neck and that in the upper extremity were 5 and 5, respectively. As we suspected cervical spondylosis, radiographical examinations were performed. X-ray images revealed foraminal stenosis of the left C4/5 and right C6/7 (Figure 1A, 1B). Cervical canal stenosis from C3 to C7 was also observed on magnetic resonance imaging (Figure 1C). Computed tomography revealed osteophytes causing the foraminal stenos of the left C5 and right C7 (Figure 1D-1F).

**Figure 2:**
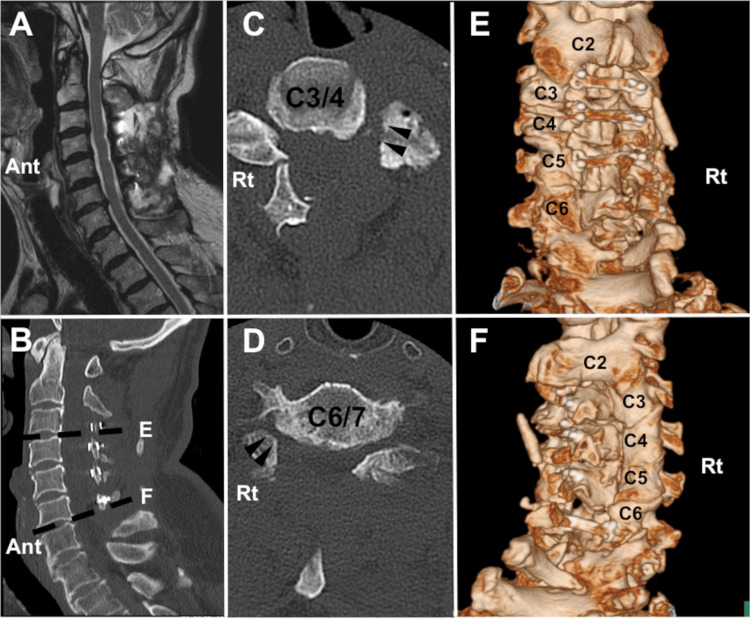
Radiographic images after the operation (case 1). Cervical canal stenosis was resolved after the operation (A: sagittal magnetic resonance image, B: sagittal computed tomography image). Posterior decompression is achieved after foraminotomy in the left C3/4 (C) and right C6/7 (D) levels. Left laminoplasty is performed from C3 to C5 (E), while right laminoplasty is performed at the level of C6 (F) (Ant: anterior, Rt: right)

The patient’s symptoms were thought to have resulted from the myelopathy and radiculopathy of the left C4 and right C7. To resolve the coexistent myelopathy and radiculopathy in a single session, laminoplasty from C3 to C6 and foraminotomy of the left C4 and right C7 were performed. Laminae from C3 to C5 were elevated on the left side, while the lamina of C6 was elevated on the right side (Figure 2).

The patient was discharged 11 days after surgery and was followed in an outpatient clinic. Three months after the operation, the VAS scores of the pain in the neck and that in the upper extremity decreased to 4 and 0, respectively.

Case 2

**Figure 3:**
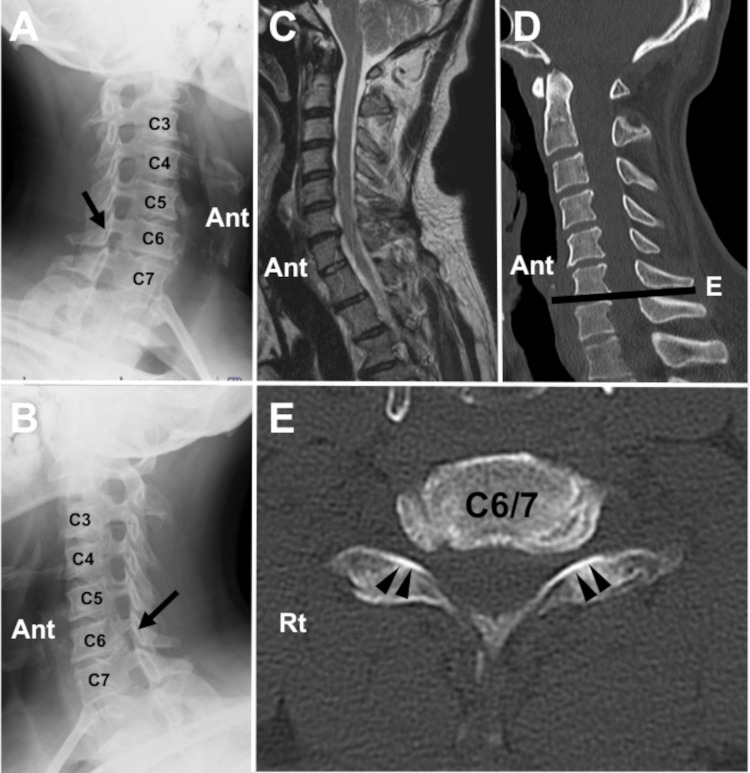
Preoperative radiographic images (Case 2). Oblique X-ray images reveal the stenosis in the bilateral C6/7 foramen (A, B, black arrows). A sagittal magnetic resonance image shows cervical spinal canal stenosis (C). (D-E) Computed tomography images disclose osteophytes forming the foraminal stenosis in the bilateral C6/7 foramen (black arrowheads) (Ant: anterior, Rt: right).

A left-handed 43-year-old man presented to our neurosurgery department complaining of pain in the neck. The patient was not able to sleep in the supine position because of the neck pain. He also mentioned pain radiating to the bilateral superior border of the scapula and bilateral upper extremity. The region of the sensory disturbance corresponded to the dermatome of bilateral C7. The pain was more severe on the right side than on the left. The VAS scores of the pain in the neck and that in the upper extremity were 8 and 8, respectively. X-ray images revealed foraminal stenosis of the bilateral C6/7 (Figure 3A, 3B). Cervical canal stenosis was observed on magnetic resonance imaging (Figure 3C). Computed tomography revealed osteophytes causing the foraminal stenosis of the bilateral C7 (Figure 3D, 3E).

**Figure 4:**
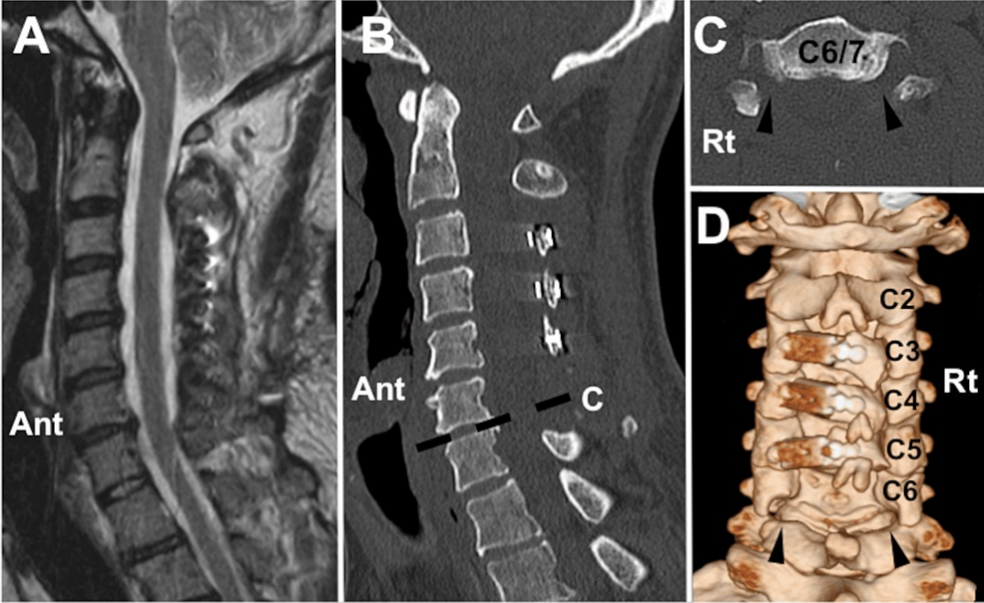
Radiographic images after the operation (case 2). Cervical canal stenosis was resolved after the operation (A: sagittal magnetic resonance image, B: sagittal computed tomography image). Posterior decompression is achieved after laminectomy and foraminotomy in the bilateral C6/7 levels (C). Left laminoplasty from C3 to C5 is combined with laminectomy of C6 and foraminotomy of the bilateral C6/7 levels (Ant: anterior, Rt: right)

The patient’s symptoms were thought to have resulted from the myelopathy and radiculopathy of the bilateral C7. Laminoplasty from C3 to C5 and laminectomy of C6 were followed by the foraminotomy of the bilateral C7 (Figure 4).

Six months after the operation, the VAS scores of the pain in the neck and that in the upper extremity decreased to 4 and 1, respectively. The patient was happy to be able to sleep in the supine position without neck pain.

## Discussion

We describe two cases of cervical spondylosis causing myelopathy and radiculopathy. Based on the preoperative neurological examination, the lesions irritating the patients were identified. A tailor-made combination of laminoplasty, laminectomy, and foraminotomy for the targeted lesions resolved myelopathy and radiculopathy of the patients successfully. The open side of laminectomy was also modified to adequately perform foraminotomy for the left C3/4 and right C6/7 levels. Our surgical approach can resolve coexistent myelopathy and radiculopathy simultaneously.

For concurrent cervical myelopathy and radiculopathy, a two-staged anterior and posterior approach can be considered [5]. A reason for this strategy was that sufficient decompression could not be achieved only with cervical laminoplasty for radiculopathy. To overcome this weakness of cervical laminoplasty, anterior cervical discectomy, and fusion were performed for radiculopathy. However, there are several postoperative complications concerning this approach, such as dysphagia, recurrent laryngeal nerve palsy, loss of spinal mobility, and increased adjacent segmental disease risk [7,8]. Furthermore, as there are important organs, including the carotid artery, jugular veins, trachea, and esophagus, along the pathway of anterior cervical discectomy and fusion to the cervical vertebrae, complications related to those organs can be fatal.

Foraminotomy (posterior approach) has been reported to be effective for cervical radiculopathy [9]. This approach showed no difference in clinical outcome, complication rate, and reoperation rate compared to those of anterior cervical discectomy and fusion [10]. Therefore, by combining laminoplasty and foraminotomy, coexistent cervical myelopathy and radiculopathy can be approached in a single session.

Previously, we described combined laminoplasty and foraminotomy, showing its safety and effectiveness for coexistent cervical myelopathy and radiculopathy [5]. However, the previous report performed this approach only for coexistent cervical myelopathy and unilateral radiculopathy. Thus, simply combining laminoplasty and foraminotomy may have a limitation in resolving concurrent cervical myelopathy and bilateral radiculopathy.

As our patients needed surgical treatment for concurrent cervical myelopathy and bilateral radiculopathy, we performed a modified combination of laminoplasty and foraminotomy. For the first patient, the open side of laminoplasty was adjusted to perform foraminotomy appropriately. The laminae from C3 to C5 were elevated on the left side for the left C3/4 foraminotomy, while the lamina of C6 was on the right for the right C6/7 foraminotomy. In the second patient, bilateral C6/7 foraminotomy was necessary for symptomatic bilateral C7 radiculopathy. In this case, however, bilateral C6/7 foraminotomy could not have been achieved if laminectomy of C6 was performed. This is because laminectomy can only allow for foraminotomy on the open side. Therefore, we selected laminectomy of C6. Both cases were managed in a single session by appropriately approaching coexistent myelopathy and bilateral radiculopathy.

As evaluated with the VAS scores, the patient’s symptoms decreased postoperatively. This suggests the effectiveness of the tailor-made combination of laminoplasty, laminectomy, and foraminotomy for coexistent cervical myelopathy and bilateral radiculopathy. Prior to surgical treatment, attentive neurological examination is essential. Because the patients are followed relatively for a short period, a long-term follow-up is warranted to evaluate long-term surgical outcomes.

## Conclusions

Effectively combining laminoplasty, laminectomy, and foraminotomy is useful for patients with coexistent cervical myelopathy and bilateral radiculopathy. A combination of these operative procedures, as a less invasive method, can resolve cervical spondylosis in a single session. Because the surgical strategy for cervical myelopathy and radiculopathy is based on neurological examination, a cautious neurological examination should be performed preoperatively.
